# Supplementary material for: Translation, adaptation and psychometric evaluation of the German version of the Abortion Attitude Scale – A secondary analysis of a cross-sectional study among medical students
Source: PLoS One. 2026 Jan 2;21(1):e0321840. doi: 10.1371/journal.pone.0321840 (PMC12758734; doi:10.1371/journal.pone.0321840)
Supplement: S1 Appendix — (DOCX) [file pone.0321840.s001.docx]

**S1 Appendix. Details on item translation adaptation.**

Table A: Results of the translation and adaptation process of the AAS: Items of the original scale, results of the translation and adaptation process (within the study team) and results of cognitive interviews with n=10 medical students.

|  | **Original scale** | **Results of the translation process** | **Results of the adaptation process** | **Final scale after three rounds of cognitive interviews** |
| --- | --- | --- | --- | --- |
| Introduction and rating scale | This is not a test. There are no wrong or right answers to any of the statements, so just answer as honestly as you can. The statements ask you to tell how you feel about legal abortion (the voluntary removal of a human fetus from the mother during the first three months of pregnancy by a qualified medical person). Tell how you feel about each statement by circling one of the choices beside each sentence. Here is a pratice statement:  SA A SlA SlD D SD  Abortion should be legalized.  (SA = Strongly Agree; A = Agree; SlD = Slightly Agree; SlD = Slightly Disagree; D = Disagree; SD = Strongly Disagree)  Please respond to each statement and circle only one response. No one else will see your responses without permission. | Dies ist kein Test. Es gibt keine richtigen oder falschen Antworten. Bitte antworten Sie so ehrlich wie möglich. In den Aussagen geht es um Ihre Ansichten über legale Schwangerschaftsabbrüche (willentliche Entfernung eines menschlichen Fötus während der ersten drei Monate der Schwangerschaft durch eine qualifizierte medizinische Person). Bitte geben Sie an, wie sehr Sie einer Aussage zustimmen oder sie ablehnen, indem Sie eine Antwortoption ankreuzen. Hier ist ein Beispiel:  stimme vollkommen zu - stimme zu - stimme eher zu - stimme eher nicht zu - stimme nicht zu - stimme überhaupt nicht zu  Schwangerschaftsabbrüche sollten legalisiert werden.  Bitte beantworten Sie jede Aussage und wählen Sie immer nur eine Antwortmöglichkeit aus. Niemand wird Ihre Antwort ohne Erlaubnis sehen können. | oder falschen Antworten. Bitte antworten Sie so ehrlich wie möglich. In den Aussagen geht es um Ihre Ansichten über **Schwangerschaftsabbrüche während der ersten drei Monate der Schwangerschaft durch eine qualifizierte medizinische Person**. Bitte geben Sie an, wie sehr Sie einer Aussage zustimmen oder sie ablehnen, indem Sie eine Antwortoption ankreuzen. Hier ist ein Beispiel:  stimme vollkommen zu - stimme zu - stimme eher zu - stimme eher nicht zu - stimme nicht zu - stimme überhaupt nicht zu  Schwangerschaftsabbrüche sollten legalisiert werden.  Bitte beantworten Sie jede Aussage und wählen Sie immer nur eine Antwortmöglichkeit aus. Niemand wird Ihre Antwort ohne Erlaubnis sehen können. | Dies ist kein Test. Es gibt keine richtigen oder falschen Antworten. Bitte antworten Sie so ehrlich wie möglich. In den Aussagen geht es um Ihre Ansichten über **Schwangerschaftsabbrüche während der ersten drei Monate der Schwangerschaft durch eine qualifizierte medizinische Person**. Bitte geben Sie an, wie sehr Sie einer Aussage zustimmen oder sie ablehnen, indem Sie eine Antwortoption ankreuzen. Hier ist ein Beispiel:  stimme vollkommen zu - stimme zu - stimme eher zu - stimme eher nicht zu - stimme nicht zu - stimme überhaupt nicht zu  Schwangerschaftsabbrüche sollten legalisiert werden.  Bitte beantworten Sie jede Aussage und wählen Sie immer nur eine Antwortmöglichkeit aus. Niemand wird Ihre Antwort ohne Erlaubnis sehen können. |
| Item 1 | The supreme court should strike down legal abortions in the United States. | Der oberste Gerichtshof sollte legale Schwangerschaftsabbrüche in den Vereinigten Staaten verbieten. | **In Deutschland** sollten Schwangerschaftsabbrüche **unter allen Umständen** verboten werden. | **In Deutschland** sollten Schwangerschaftsabbrüche **unter allen Umständen** verboten werden. |
| Item 2 | Abortion is a good way of solving an unwanted pregnancy. | Ein Schwangerschaftsabbruch ist eine gute Möglichkeit, eine ungewollte Schwangerschaft zu beenden. | *No adaptation of the translated version.* | Ein Schwangerschaftsabbruch ist eine gute Möglichkeit, eine ungewollte Schwangerschaften zu beenden. |
| Item 3 | A mother should feel obligated to bear a child she has conceived. | Eine Mutter sollte sich verpflichtet fühlen, das Kind zu gebären, das sie gezeugt hat. | **Eine Person** sollte sich verpflichtet fühlen, das Kind zu gebären, das sie gezeugt hat. | **Eine Person** sollte sich verpflichtet fühlen, das Kind zu gebären, **das gezeugt wurde**. |
| Item 4 | Abortion is wrong no matter what the circumstances are. | Schwangerschaftsabbrüche sind unter allen Umständen falsch. | *No adaptation of the translated version.* | Schwangerschaftsabbrüche sind unter allen Umständen falsch. |
| Item 5 | A fetus is not a person until it can live outside its mother’s body. | Ein Fötus ist so solange keine Person, bis er außerhalb des Körpers der Mutter leben kann. | Version 1: Ein Fötus ist so **solange keine** Person, bis er außerhalb des Körpers der **schwangeren Person** leben kann.  Version 2: Ein Fötus ist **erst eine** Person, wenn er außerhalb des Körpers der **schwangeren Person** leben kann. | Ein Fötus **ist erst eine** Person, wenn er außerhalb des Körpers der **schwangeren Person** leben kann. (Version 2) |
| Item 6 | The decision to have an abortion should be the pregnant mother’s. | Die Entscheidung dafür, einen Schwangerschaftsabbruch durchführen zu lassen, sollte bei der schwangeren Mutter liegen. | Version 1: Die Entscheidung **für einen Schwangerschaftsabbruch** sollte bei der **schwangeren Person** liegen.  Version 2: Die Entscheidung dafür, einen Schwangerschaftsabbruch durchführen zu lassen, sollte bei der **schwangeren Person** liegen. | Die Entscheidung für **einen Schwangerschaftsabbruch** sollte bei der **schwangeren Person** liegen. (Version 1) |
| Item7 | Every conceived child has the right to be born. | Jedes gezeugte Kind hat das Recht, geboren zu werden. | *No adaptation of the translated version.* | Jedes gezeugte Kind hat das Recht, geboren zu werden. |
| Item 8 | A pregnant female not wanting to have a child should be encouraged to have an abortion. | Eine schwangere Frau, die kein Kind haben möchte, sollte zu einem Schwangerschaftsabbruch ermutigt werden. | Eine **schwangere Person**, die kein Kind haben möchte, sollte zu einem Schwangerschaftsabbruch ermutigt werden. | Eine schwangere Person, die kein Kind haben möchte, sollte zu einem Schwangerschaftsabbruch ermutigt werden. |
| Item 9 | Abortion should be considered killing a person. | Ein Schwangerschaftsabbruch sollte als Tötung eines Menschen betrachtet werden. | Version 1: Ein Schwangerschaftsabbruch sollte als **Tötung eines Menschen** betrachtet werden.  Version 2: Ein Schwangerschaftsabbruch sollte **als Mord** gelten. | Ein Schwangerschaftsabbruch sollte als **Tötung eines Menschen** **gelten**. (new version) |
| Item 10 | People should not look down on those who choose to have abortions. | Menschen sollten nicht auf diejenigen herabschauen, die sich für einen Schwangerschaftsabbruch entscheiden. | Version 1: Menschen sollten nicht auf diejenigen herabschauen, die sich für **einen Schwangerschaftsabbruch** entscheiden.  Version 2: Menschen sollten nicht auf diejenigen herabschauen, die sich für **Schwangerschaftsabbrüche** entscheiden. | Menschen sollten nicht auf diejenigen herabschauen, die sich für **einen Schwangerschaftsabbruch** entscheiden. (Version 1) |
| Item 11 | Abortion should be an available alternative for unmarried, pregnant teenagers. | Ein Schwangerschaftsabbruch sollte eine verfügbare Alternative für unverheiratete, schwangere Teenager sein. | Version 1: Ein Schwangerschaftsabbruch sollte für schwangere Teenager **leicht verfügbar** sein.  Version 2: Schwangerschaftsabbrüche sollten eine **mögliche Alternative** für unverheiratete, schwangere Jugendliche sein. | Ein Schwangerschaftsabbruch sollte **eine leicht zugängliche Alternative** für schwangere **Minderjährige** sein. (new version) |
| Item 12 | Persons should not have the power over the life or death of a fetus. | Personen sollten nicht die Macht über das Leben oder den Tod eines Fötus haben. | **Niemand** sollte die Macht über das Leben oder den Tod eines Fötus haben. | Niemand sollte die Macht über das Leben oder den Tod eines Fötus haben. |
| Item 13 | Unwanted children should not be brought into the world. | Ungewollte Kinder sollten nicht in die Welt gesetzt werden. | *No adaptation of the translated version.* | Ungewollte Kinder sollten nicht **auf die Welt gebracht** werden. |
| Item 14 | A fetus should be considered a person at the moment of conception. | Ein Fötus sollte ab dem Zeitpunkt der Empfängnis als Person betrachtet werden. | *No adaptation of the translated version.* | Ein Fötus sollte ab dem Zeitpunkt der Empfängnis als Person betrachtet werden. |

Table B: Demographic data of participants of cognitive interviews (n=10)

|  |  | **n** | **%** |
| --- | --- | --- | --- |
| Age | 22-25 years | 6 | 60.0 |
|  | 26-28 years | 4 | 40.0 |
| Gender | Female | 7 | 70.0 |
|  | Male | 3 | 30.0 |
| Desired field of medical specialization | Dermatology | 1 | 10.0 |
|  | Gynecology | 4 | 40.0 |
|  | Internal medicine | 2 | 20.0 |
|  | Psychiatry / Psycho-somatics | 2 | 20.0 |
|  | Not decided yet | 1 | 10.0 |
| Religion | None | 6 | 60.0 |
|  | Protestant | 4 | 40.0 |
| Place of origin | Baden-Württemberg | 2 | 20.0 |
|  | Hamburg | 1 | 10.0 |
|  | Hessen | 1 | 10.0 |
|  | Lower Saxony | 2 | 20.0 |
|  | North Rhine-Westphalia | 1 | 10.0 |
|  | Saxony | 1 | 10.0 |
|  | Schleswig-Holstein | 2 | 20.0 |
| Place of living | Baden-Württemberg | 1 | 10.0 |
|  | Hamburg | 7 | 70.0 |
|  | Saxony-Anhalt | 2 | 20.0 |
| Migrant background | Yes | 1 | 10.0 |
|  | No | 9 | 90.0 |
