# Supplementary material for: Translation, adaptation and psychometric evaluation of the German version of the Abortion Attitude Scale – A secondary analysis of a cross-sectional study among medical students
Source: PLoS One. 2026 Jan 2;21(1):e0321840. doi: 10.1371/journal.pone.0321840 (PMC12758734; doi:10.1371/journal.pone.0321840)
Supplement: S2 Appendix — (DOCX) [file pone.0321840.s002.docx]

**S2 Appendix. Further demographic data and inter-item correlations.**

Table A: Further demographic characteristics of participants (n=305 medical students).

|  |  | **n** | **%** |
| --- | --- | --- | --- |
| Own country of birth | Germany | 226 | 74.1 |
|  | Others^a^ | 8 | 2.6 |
|  | Missings | 71 | 23.3 |
| Parents country of birth^#^ | Germany | 204 | 66.9 |
|  | Mother born in other country than Germany^b^ | 15 | 4.9 |
|  | Father born in other country than Germany^c^ | 15 | 4.9 |
|  | Missings | 71 | 23.3 |
| State of origin | Baden-Württemberg | 20 | 6.6 |
|  | Bavaria | 17 | 5.6 |
|  | Berlin | 5 | 1.6 |
|  | Brandenburg | 4 | 1.3 |
|  | Bremen | 3 | 1.0 |
|  | Hamburg | 2 | 0.7 |
|  | Hesse | 5 | 1.6 |
|  | Mecklenburg-West Pomerania | 2 | 0.7 |
|  | Lower Saxony | 28 | 9.2 |
|  | North Rhine-Westphalia | 116 | 38.0 |
|  | Rhineland-Palatinate | 6 | 2.0 |
|  | Saxony | 12 | 3.9 |
|  | Saxony-Anhalt | 1 | 0.3 |
|  | Schleswig-Holstein | 7 | 2.3 |
|  | Thuringa | 4 | 1.3 |
|  | Missings | 73 | 23.9 |
| Hometown size | less than 5,000 inhabitants (rural municipality) | 39 | 12.8 |
|  | 5,000 to under 20,000 inhabitants (small town) | 46 | 15.1 |
|  | 20,000 to under 100,000 inhabitants (medium-sized town) | 55 | 18.0 |
|  | over 100,000 inhabitants (large city) | 94 | 30.8 |
|  | Missings | 71 | 23.3 |
| Mother Tongue | German | 228 | 74.8 |
|  | Other^d^ | 6 | 2.0 |
|  | Missing | 23.3 |  |
| Pregnancies | 0 | 213 | 69.8 |
|  | 1 | 15 | 4.9 |
|  | 2 | 1 | 0.3 |
|  | 5 | 1 | 0.3 |
|  | Missings | 75 | 24.6 |
| Children | 0 | 223 | 73.1 |
|  | 1 | 5 | 1.6 |
|  | 2 | 2 | 0.7 |
|  | 4 | 1 | 0.3 |
| Confession | None | 110 | 36.1 |
|  | Roman Catholic | 57 | 18.7 |
|  | Protestant | 55 | 18.0 |
|  | Islamic | 5 | 1.6 |
|  | Jewish | 1 | 0.3 |
|  | Other^e^ | 6 | 2.0 |
|  | Missings | 71 | 23.3 |

Note: ^a^ Ecuador, Luxembourg, New Haven, Russia, Switzerland, Spain (n=2), USA; ^b^ Bosnia and Herzogovina, Chile, Ecuador, France, Ireland, Italy, Kazakhstan, Poland (n=2), Russia, Thailand, Turkey (n=2); ^c^ Afganisthan, Greece, Chile, Denmark, France, Yugoslavia, Kazakhstan, Kirgizitan, Luxembourg, Austria, Poland, Rumania, Switzerland, Taiwan, Vietnam, d German-French, Greek, Italy and German, Luxembourgish, Spanish, Türkish; ^e^ Buddhism, Christian, Free christian, Greek orthodox; all n=1 unless otherwise specified

Table B: Inter-item correlations of the 14-item German AAS. Recommended range >0.3.

|  | Item 1 | Item 2 | Item 3 | Item 4 | Item 5 | Item 6 | Item 7 | Item 8 | Item 9 | Item 10 | Item 11 | Item 12 | Item 13 | Item 14 |
| --- | --- | --- | --- | --- | --- | --- | --- | --- | --- | --- | --- | --- | --- | --- |
| Item 1 | 1 |  |  |  |  |  |  |  |  |  |  |  |  |  |
| Item 2 | .482 | 1 |  |  |  |  |  |  |  |  |  |  |  |  |
| Item 3 | .439 | .614 | 1 |  |  |  |  |  |  |  |  |  |  |  |
| Item 4 | .493 | .464 | .450 | 1 |  |  |  |  |  |  |  |  |  |  |
| Item 5 | .260 | .404 | .427 | .238 | 1 |  |  |  |  |  |  |  |  |  |
| Item 6 | .431 | .536 | .491 | .411 | .416 | 1 |  |  |  |  |  |  |  |  |
| Item 7 | .431 | .601 | .627 | .454 | .500 | .480 | 1 |  |  |  |  |  |  |  |
| Item 8 | .280 | .386 | .280 | .153 | .330 | .319 | .257 | 1 |  |  |  |  |  |  |
| Item 9 | .536 | .607 | .665 | .507 | .477 | .589 | .678 | .338 | 1 |  |  |  |  |  |
| Item 10 | .129 | .222 | .251 | .139 | .230 | .307 | .194 | .174 | .278 | 1 |  |  |  |  |
| Item 11 | .375 | .533 | .432 | .337 | .465 | .411 | .529 | .411 | .470 | .180 | 1 |  |  |  |
| Item 12 | .499 | .585 | .509 | .414 | .449 | .505 | .681 | .298 | .634 | .195 | .530 | 1 |  |  |
| Item 13 | .313 | .406 | .429 | .298 | .405 | .389 | .418 | .522 | .402 | .141 | .453 | .312 | 1 |  |
| Item 14 | .351 | .479 | .510 | .396 | .540 | .441 | .653 | .301 | .579 | .249 | .448 | .570 | .372 | 1 |
