# Supplementary material for: Translation, adaptation and psychometric evaluation of the German version of the Abortion Attitude Scale – A secondary analysis of a cross-sectional study among medical students
Source: PLoS One. 2026 Jan 2;21(1):e0321840. doi: 10.1371/journal.pone.0321840 (PMC12758734; doi:10.1371/journal.pone.0321840)
Supplement: S3 Appendix — (DOCX) [file pone.0321840.s003.docx]

**S3 Appendix. Checklist for reporting standards.** Author’s guideline for Scale Development and validation results by Cabrera-Nguyen.

|  | **Guidelines by Cabrera-Nguyen [1]** | **Transfer to our study** |
| --- | --- | --- |
| 1 | Precisely define the target construct. | See page 5 (Introduction section) |
| 2 | Justify the need for your new measure. For example, if measures of the construct exist in the literature, explain the value added by your new scale. How might the new measure enhance the substantive knowledge base or social work practice? | See page 3 to 5 (Introduction section) |
| 3 | Indicate that you have submitted your initial pool of items to expert review (Worthington & Whittaker, 2006). Report (a) the number of items in the preliminary pool; (b) the number of expert reviewers and their qualifications; and (c) any major changes to your initial item pool following the review (e.g., a substantial decrease in the number of items, changes to the original item response format, overhaul of item pool due to experts’ assessment regarding content validity). | See page 7 and 8 (Methods section: Translation, Assessment of comprehensibility) and page 9 to11 (Results section: Translation, Assessment of comprehensibility) |
| 4 | Report the name and version of the statistical software package used for all analyses. | See page 9 (Methods section: Psychometric evaluation) |
| 5 | Identify and justify the sampling strategy (e.g., convenience, snowball) and sampling frame. Report standard sample demographic characteristics as well as other salient sample characteristics. | See page 7 and 8 (Methods section: Assessment of comprehensibility, Psychometric evaluation) and page 10 to 13 (Results section: Assessment of comprehensibility, Psychometric evaluation) and S1 Appendix |
| 6 | Discuss relevant data preparation and screening procedures. For instance, do the data meet the appropriate assumptions for factor analysis? If not, what actions were taken? Report tests of factorability if appropriate (e.g., report Bartlett’s test of sphericity). | See page 16 (Results section: Psychometric evaluation) |
| 7 | Provide all dates of data collection. | See page 9 to 17 (Results section) |
| 8 | Avoid use of principal components analysis (PCA) as a precursor to CFA (Costello & Osborne, 2005; Worthington & Whittaker, 2006). Instead, start with EFA to assess the underlying factor structure and refine the item pool. EFA should be followed by CFA using a different sample (or samples) to evaluate the EFA-informed a priori theory about the measure’s factor-structure and psychometric properties. (Costello & Osborne, 2005; Henson & Roberts, 2006; Worthington & Whittaker, 2006). For CFA, authors should specify an a priori hypothesized model and a priori competing models (Jackson, Gillaspy, & Purc-Stephenson, 2009). | An a priory hypothesized model for CFA was specified based on the original measures structure. For more information see page 16 and 17 (Results section). |
| 9 | Guidelines for reporting EFA results. | |
|  | How large is a sample? One common rule of thumb is to ensure a person-to-item ratio of 10:1. Another rule of thumb is that N= 300 is usually acceptable (Worthington & Whittaker, 2006). However, some researchers have criticized these sample size rules of thumb, noting the appropriate sample size is dependent on the features of the gathered data. These researchers recommend obtaining the largest possible sample because the adequacy of the sample size cannot be determined until after the data have been analyzed (Henson & Roberts, 2006). | Not applicable (see point 8) |
|  | Run EFA . . . or not. Run a preliminary EFA to determine if further data collection is required based on the following criteria: (a) If communalities are greater than .50 or there are 10:1 items per factor with factor loadings of roughly .4, then a sample size of 150 to 200 is likely to be adequate; (b) If communalities are all at least .60 or there are a minimum of 4:1 items per factor with factor loadings above .6, then even smaller sample sizes may suffice; (Worthington & Whittaker, 2006). Report if additional data collection was necessary due to inadequate sample size. If so, report the new participants’ sociodemographic characteristics and test for differences between groups using standard statistical procedures (e.g., t-tests). | Not applicable (see point 8) |
|  | Give EFA details. Report the specific rotation strategy used (e.g. varimax, geomin). Justify the decision to use an orthogonal or oblique solution. One recommendation is to always begin with an oblique rotation, empirically assess factor intercorrelations, and report them before deciding upon a final rotation solution (Henson & Roberts, 2006; Worthington &Whittaker, 2006). Some researchers argue oblique rotation is always the best approach because (a) factor intercorrelations are the norm in social sciences and (b) both approaches yield the same result if the factors happen to be uncorrelated (Costello & Osborne, 2005). Conversely, other researchers contend that orthogonal rotation is preferable because fewer parameters are estimated—orthogonal rotation is more parsimonious and amenable to replication (Henson &Roberts, 2006). Similarly, some researchers warn against relying on a statistical software package’s default settings to determine the appropriate type of oblique rotation (Henson & Roberts, 2006; Worthington &Whittaker, 2006). Others state that doing so is fine (Costello & Osborne, 2005, p.3). Given the lack of consensus, it is probably best to describe what you do and defend your approach on substantive grounds, if possible. | Not applicable (see point 8) |
|  | Report the whole factor pattern/structure. Always report the whole factor pattern/structure matrix, including all of the items in the analysis. It is recommended that authors report this information in a chart following the example provided by Henson and Roberts (2006) on page 411. | Not applicable (see point 8) |
|  | Criteria for deleting (crossloaded) items. Report any deleted items and the criteria used for deletion. Crossloading items with values ≥ .32 on at least two factors should generally be candidates for deletion, especially if there are other items with factor loadings of .50 or greater (Costello & Osborne,2005). Rerun the EFA each time an item is deleted. | Not applicable (see point 8) |
|  | Criteria for number of factors. Report the number of factors retained and justify this decision using multiple criteria (eigenvalue > 1, scree test, parallel analysis, rejection of a factor with fewer than 3 items, etc). Reporting the eigenvalue > 1 rule alone is inadequate because it has been shown to among the least accurate criteria for assessing factor retention (Costello & Osborne, 2005; Henson & Roberts, 2006) | Not applicable (see point 8) |
|  | Explained variance. Report the variance explained by the factors. | Not applicable (see point 8) |
|  | In general, describe your decisions. | Not applicable (see point 8) |
| 10 | Guidelines for reporting CFA results. | |
|  | Describe and justify the theoretical model. Report hypothesized factor structure. Provide theoretical and empirical justification (e.g., results of preliminary EFAs) for your hypothesis. In addition, report a priori competing models. | See page 8 and 9 (Methods section: Psychometric evaluation) |
|  | Describe the parameterization. Provide a comprehensive description of the a priori parameter specification. Identify fixed parameters, free parameters, and constrained parameters. For example, indicate if you freed the errors of any items to correlate. | One factor loading was constrained to equal 1, the corresponding intercept was constrained equal to zero. The other factor loadings and intercepts were estimated. Errors of items were not freed to correlate. |
|  | Include a figure. Include a figure of each CFA model being tested using Kline’s (2005) graphical conventions if feasible. | We decided to not include a figure but only a table containing fit indices. |
|  | Identification. Demonstrate model identification (e.g., df > 0; scaling of factors; assess and report the ―t-rule; the two-indicator rule). Necessary and sufficient conditions for model identification may vary for certain types of CFA models. When in doubt, authors should consult Brown’s (2006) CFA textor Kline’s (2005) SEM text for guidance. | See page 16 and 17 (Results section: Psychometric evaluation) |
|  | Select an estimator based on distributional patterns and assumptions. Report the estimator used (e.g., ML, WLSMV) and justify your choice based on distributional assumptions. It is not appropriate to report that you relied on your statistical software’s default setting. | See page 9 (Methods section: Psychometric evaluation) |
|  | Use multiple fit indices. After estimating a model, always report multiple fit indices (e.g., model X2, df, p, CFI/TLI, RMSEA, SRMR). Report all appropriate fit indices, not just those favorable to your hypotheses (Jackson et al., 2009). For example, do not report acceptable CFI and TLI scores while omitting a relevant fit index with a subobtimal value. | See page 9 (Methods section: Psychometric evaluation) and page 16 and 17 (Results section: Psychometric evaluation) |
|  | What is acceptable fit? For model fit indices, authors should generally use the cut-off values recommended by Hu and Bentler (1999) and endorsed by Brown (2006), assuming ML estimation: a.CFI/TLI ≥ .95 b. RMSEA ≤ .06 c. SRMR ≤ .08 | See page 9 (Methods section: Psychometric evaluation) |
|  | Localized strain? When reporting model fit, include an assessment for localized areas of strain by examining standardized residuals. Standardized residuals greater than 1.96 (for p< .05) indicate areas of strain (Harrington, 2009). Report the absence of localized strain, if appropriate; otherwise, note localized areas of strain by reporting the relevant standardized residuals. | Standardized residuals do not indicate localized strains. |
|  | Parameter estimates and SEs. When reporting factor loadings and other parameter estimates, always report the unstandardized estimates, their p values, and the standard errors. In addition, include the standardized estimates when appropriate. Be sure to report all parameter estimates, even those that are non significant (Brown, 2006; Jackson et al., 2009). | See page 16 and 17 (Results section: Psychometric evaluation) |
|  | Assessing the validity of the factor solution. Comment on the new measure’s convergent and discriminant validity based on parameter estimates. For instance, factor correlations ≥ .80 may indicate poor discriminant validity (Brown, 2006). In addition, strong factor loadings that do not crossload may indicate good convergent validity. One rule of thumb is that factor loadings < .40 are weak and factor loadings ≥ .60 are strong (Garson, 2010). | See page 16 and 17 (Results section: Psychometric evaluation) |
|  | Other measures. Report squared multiple correlations and comment on the measure’s reliability (e.g., report Raykov’s Rho if appropriate) | See page 16 and 17 (Results section: Psychometric evaluation) |
|  | Respecification: Caution! Report any post-hoc respecifications to improve model fit based on modification indices. Justify the respecifications on theoretical or conceptual grounds (Jackson et al., 2009). Respecification to allow for correlated errors is not supportable without strong pragmatic justification(e.g., items contain similar words or phrases). Note that respecification precludes comparing the model with your a priori specified competing models. Report improvements in appropriate model fit indices for respecified models (e.g., chi-square difference test) | No respecifications to improve model fit were applied. |
| 10 | Describe the matrix (or matrices) you analysed (e.g., covariance, correlation). Include matrices in the manuscript if feasible; otherwise, indicate these data are available upon request. | See page 16 and 17 (Results section: Psychometric evaluation) |
| 11 | Report the amount of missing data and describe how missing data were handled. For a review of practices for handling missing data, see Sterne and colleagues (2009), Rose and Fraser (2008), and Horton and Kleinman (2007).Provide a rationale for your approach to handling missing data. Authors are encouraged to consider using multiple imputation or model estimation with full-information maximum likelihood (FIML; Rose & Fraser, 2008). | See page 9 (Methods section: Psychometric evaluation) and page 16 and 17 (Results section: Psychometric evaluation) |
| 12 | Compare your CFA model with the alternative or competing models. Do competing models fit the data better or worse than your model (e.g., does your four-factor model of acculturation fit the data better than a two-factor model or a one-factor model)? Identify the preferable model based on appropriate fit statistics (e.g. chi-square difference test for nested models, Akaike information criterion for non-nested models), parsimony, and relevant theory | See page 18 (Discussions sections) |
| 13 | Include your scale (items and response options) in an appendix. | See Table 1 and S1 Appendix |
| 14 | Report how methodological limitations may have impacted findings regarding your measure’s psychometric properties (e.g., note potential repercussions of suboptimal sampling techniques, discuss implications of using listwise deletion to handle missing data instead of multiple imputation or FIML). | See page 19 and 20 (Discussions sections) |
| 15 | Discuss directions for future research (e.g., if appropriate, testing your scale for measurement invariance by conducting CFA on different populations). | See page 19 (Discussions sections) |

Cabrera-Nguyen P. Author Guidelines for Reporting Scale Development and Validation Results. *J Soc Social Work Res*. University of Chicago PressChicago, IL 2010;1:99–103.
