# Supplementary material for: Translation, adaptation and psychometric evaluation of the German version of the Abortion Attitude Scale – A secondary analysis of a cross-sectional study among medical students
Source: PLoS One. 2026 Jan 2;21(1):e0321840. doi: 10.1371/journal.pone.0321840 (PMC12758734; doi:10.1371/journal.pone.0321840)
Supplement: Attachment — Submitted filename: Lindig et al._AAS_PLOS ONE_response to reviewers_final.pdf [file pone.0321840.s005.pdf]

PONE-D-25-12818
Translation, adaptation and psychometric evaluation of the German version of the Abortion Attitude Scale – a secondary analysis of a cross-sectional study among medical students
PLOS ONE

Journal requirements / Comments of the editor:
When submitting your revision, we need you to address these additional requirements.

1. Please ensure that your manuscript meets PLOS ONE's style requirements, including those for file naming.
   ➔ Dear editors, thank you for this suggestion. We carefully checked the style requirements at the PLOS ONE webpage and the manuscript and supporting information. We made slight changes to the naming of appendix files and the headings of those files. You find the revised files attached. We could not find any other points, where we did not meet style requirements. If there are any remaining aspects where we have not met the requirements, we would greatly appreciate further specific guidance and would be happy to make the necessary improvements.

2. Thank you for stating the following financial disclosure:
   "This study was planned and conducted as part of the study "Person-centeredness in healthcare and support services for women with unwanted pregnancy" (CarePreg), which was funded by the German Federal Ministry of Health (Bundesministerium für Gesundheit, BMG) with the grant number 2520FSB113. Dr. Jördis Zill (Co-author) was principal investigator of the CarePreg Study and received the funding."
   Please state what role the funders took in the study. If the funders had no role, please state: "The funders had no role in study design, data collection and analysis, decision to publish, or preparation of the manuscript."
   If this statement is not correct you must amend it as needed. Please include this amended Role of Funder statement in your cover letter; we will change the online submission form on your behalf.
   ➔ We included the statement regarding funding in the cover letter and deleted it from the main manuscript: *"This study was planned and conducted as part of the study "Person-centeredness in healthcare and support services for women with unwanted pregnancy" (CarePreg), which was funded by the German Federal Ministry of Health (Bundesministerium für Gesundheit, BMG) with the grant number: 2520FSB113. Dr. Jördis Zill (co-author) was principal investigator of the CarePreg Study and received the funding. The funders had no role in study design, data collection and analysis, decision to publish, or preparation of the manuscript."*

3. Thank you for stating the following in the Funding Section of your manuscript:
   "This study was planned and conducted as part of the study "Person-centeredness in healthcare and support services for women with unwanted pregnancy" (CarePreg), which was funded by the German Federal Ministry of Health (Bundesministerium für Gesundheit, BMG) with the grant number 2520FSB113."
   We note that you have provided funding information that is currently declared in your Funding Statement. However, funding information should not appear in the Acknowledgments section or other areas of your manuscript. We will only publish funding information present in the Funding Statement section of the online submission form.
   Please remove any funding-related text from the manuscript and let us know how you would like to update your Funding Statement. Currently, your Funding Statement reads as follows:
   "This study was planned and conducted as part of the study "Person-centeredness in healthcare and support services for women with unwanted pregnancy" (CarePreg), which was funded by the German Federal Ministry of Health (Bundesministerium für Gesundheit, BMG) with the grant number 2520FSB113. Dr. Jördis Zill (Co-author) was principal investigator of the CarePreg Study and received the funding."
   Please include your amended statements within your cover letter; we will change the online submission form on your behalf.

- ➔ Thank you very much for this information and we have to apologize for this mistake. We deleted the "Funding" subsection in the DECLARATIONS section and revised the according paragraph in the Cover Letter as follows: *"This study was planned and conducted as part of the study "Person-centeredness in healthcare and support services for women with unwanted pregnancy" (CarePreg), which was funded by the German Federal Ministry of Health (Bundesministerium für Gesundheit, BMG) with the grant number: 2520FSB113. Dr. Jördis Zill (co-author) was principal investigator of the CarePreg Study and received the funding. The funders had no role in study design, data collection and analysis, decision to publish, or preparation of the manuscript."*
- ➔ It would be very helpful if you could update the statement in the online submission form accordingly.

4. In the online submission form you indicate that your data is not available for proprietary reasons and have provided a contact point for accessing this data. Please note that your current contact point is a co-author on this manuscript. According to our Data Policy, the contact point must not be an author on the manuscript and must be an institutional contact, ideally not an individual. Please revise your data statement to a non-author institutional point of contact, such as a data access or ethics committee, and send this to us via return email.
   - ➔ According to your data policy suggestions, we shared our data in an appropriate data repository. The whole data set is now published on the OSF data repository platform (Center for Open Science). The project ID is https://doi.org/10.17605/OSF.IO/MQ3SN and the view-only link to the project is https://osf.io/mq3sn/overview?view_only=2d23b7900c774696a0f29b5a660e14b1. So I will send the following information per return email to be included in the online submission form: "*A complete data set of this studies data is available within a project on the OSF data repository and thus accessible via the following doi: https://doi.org/10.17605/OSF.IO/MQ3SN or the following link: https://osf.io/mq3sn/overview?view_only=2d23b7900c774696a0f29b5a660e14b1.*" Please state, if there are any templates of your journal, I should use for this statement.
   - ➔ Furthermore, we revised the subsection "Availability of data and materials" in the DECLARATIONS section accordingly: *"The dataset collected and analyzed during this study is available from the OSF data repository (https://doi.org/10.17605/OSF.IO/MQ3SN)."* Please state, if we should shift this subsection "Availability of data and materials" to another section in the manuscript or if there is a specific template, we should use to formulate this paragraph.

5. Please also include contact information for the third party organization, and please include the full citation of where the data can be found.
   - ➔ Please see our comment on your suggestion #4. In the revised "Availability of data and materials" section we provided the doi and the link to access the data set on OSF.

6. Your ethics statement should only appear in the Methods section of your manuscript. If your ethics statement is written in any section besides the Methods, please move it to the Methods section and delete it from any other section. Please ensure that your ethics statement is included in your manuscript, as the ethics statement entered into the online submission form will not be published alongside your manuscript.
   - ➔ Thank you very much for this clarification. We shifted the subsection on ethics approval and consent to participate to the METHODS section and deleted it in the DECLARATIONS section. We also made small adjustments to the ethics statement to improve language.

7. If the reviewer comments include a recommendation to cite specific previously published works, please review and evaluate these publications to determine whether they are relevant and should be cited. There is no requirement to cite these works unless the editor has indicated otherwise.
   - ➔ Thank you very much for this suggestion, which we will implement accordingly.

Comments of the reviewers

Reviewer #1

8. The article has excellent practices on writing, argumentation and data analysis. I provide below some suggestions for enhancing the quality of the manuscript:
   - ➔ Thank you very much for the very positive feedback on our article. We are very happy about every suggestion which strengthens the quality of our manuscript.

9. Along all pages, consider the division of in long paragraphs in smaller ones, so that reading is more fluid.
   - ➔ Thank you for this suggestion. We have implemented this and divided longer paragraphs into smaller ones throughout the whole document. The changes can be tracked and reviewed in the version with Tracked Changes.

10. Review the use of the term "factorial structure" (it should be just "factor structure").
    - ➔ Thank you very much for the detailed reading. The term "factorial structure" is of course not right in this context. We changed the term "factorial structure" to "factor structure" throughout the whole manuscript.

11. When describing the methodology of cognitive interviews, consider providing examples of questions, so that this procedure may be better understood.
    - ➔ Thank you for this important suggestion. We provided some examples of questions from the interview guide in the revised "Assessment of comprehensibility" subsection in the METHODS section: *"The interview guide included questions such as "What do you understand by abortion?", "How do you understand this statement?", "What do you understand by murder in this item?". Alternatively, if different versions of an item were presented: "Is this version easier to understand than the first version?", "Does the question mean the same thing?"."*

12. In data analysis, describe how item difficulty was calculated.
    - ➔ We elaborated on the according paragraph explaining calculation of item difficulties and revised the respective sentence to clarify on our approach: *"This involved examining response distribution, item difficulty (calculated by standardizing the mean to range from 0 to 1 by division by the maximum possible value of 5, recommended range 0.2 – 0.8) to assess floor and ceiling effects (item difficulties below 0.2 indicate a floor effect; item difficulties above 0.8 indicate a ceiling effect)."* Additionally, we added the following reference to this revised section: Döring N, Bortz J. Forschungsmethoden und evaluation. Wiesbaden: Springerverlag, 2006.

13. In the discussion, consider a shorter description of the results observed in the first paragraph, so that you can focus on implications for theory and practice in the subsequent text. The same recommendation applies to describing how you concluded for a third model of the instrument.
    - ➔ Thank you very much for this comment. We shortened the according paragraphs summarizing results of this study in the discussions' subsection "Translation, adaptation and psychometric evaluation" as follows:
      "The translation team quickly agreed on the translation of the AAS. No adaptations to the translated versions were necessary for items 2, 4, 7, 13, and 14. After minor adaptations, the introduction as well as items 1, 2, 4, 7, 8, 12, and 14 were well understood in cognitive interviews. For items 5, 6, and 10, one of two item versions was chosen, while items 3, 9, 11, and 13 were reworded based on participants' feedback. The final round of cognitive interviews confirmed that all items were comprehensible.
      The German AAS demonstrated high acceptability. Most participants indicated having high AAS sum scores and were categorized as having a strong pro or moderate pro-abortion attitude (47.87% and 36.39% respectively). Only 3.5% were categorized as holding moderate or strong anti-abortion attitudes. Ceiling effects were observed, reflecting the sample's generally positive abortion attitude."

[…]
"Item characteristics for the German AAS were satisfying for most items, except for item 10 (original: "People should not look down on those who choose to have abortions"). This item showed a low corrected item-total correlations, an unfavourable response distribution (demonstrating a ceiling effect), weak inter-item correlation, and a factor loading below the recommended threshold of 0.40 in the CFA. These results suggest that item 10 does not measure the underlying concept (50,51,69). In model 1, the a priori hypothesized one-factor structure was supported, although overall model fit indices were not fully satisfactory and item 10 showed the weakest factor loading. After removing item 10 (model 2) and additionally allowing a correlation between item 8 and 13 based on modification indices and item's content (model 3), model 3 resulted in the best model fit and acceptable factor loadings (55,56,64)."

14. In line 454, review use of commas (suggestion as follows): "it was, for example, not possible to" (...).
    - ➔ Thank you very much for the detailed reading. We changed the according sentence as follows: *"It was, for example, not possible to assess convergent or divergent validity."*

15. The reference section has more than 70 references, which may not follow the journal's guidelines - consider review.
    - ➔ Thank you very much for your suggestion. According to the journal's website, there is no limit for the number of references. We also recognized that several papers, published in PLOS ONE, included 80 references and more. Therefore, we did not deleted references from our manuscript. However, if the editors also agree to your suggestion, we are of course very open to reduce our number of references.

<u>Reviewer #2</u>

16. Line 211 reports an incorrect cut point for the KMO, indicating it as .05.
    - ➔ Thank you very much for the careful reading. This was a mistake and we changed this sentence accordingly: *"KMO value should be higher than .50 and Bartlett`s test value should be less than .05 to fulfil the criteria for calculating a factor analysis (50,51)."*

17. The study does not report the use of dimensionality testing procedures, such as parallel analysis or other recommended approaches.
    - ➔ Thank you very much for your content. Methods like parallel analysis use an explorative approach. Here, we had a prior hypothesis about the dimensionality based on the original instrument. In this case confirmatory factor analysis is more appropriate. To also make this clearer in the manuscript, we revised the section on psychometric analysis accordingly (*"Furthermore, other studies using the AAS did not analyse the factor structure of the instrument but relied on the assumption of a one-factor-structure (34,35). Thus, a previous hypothesis about the dimension of the construct is available and a CFA is therefore appropriate."*) and we added a reference to this last sentence: De Vet, H. C., Terwee, C. B., Mokkink, L. B., & Knol, D. L. (2011). *Measurement in medicine: a practical guide*. Cambridge university press. (page 72).

18. Between lines 222 and 227, there is no mention of cut-off criteria for fit indexes or omega.
    - ➔ Thank you very much for the detailed reading. The recommended cut-off criteria are specified in table 6. However, since they were not included in the methods section so far, we also included them in the revised paragraph in the "Psychometric data analyses" subsection:
      "To evaluate model fit of each model, a range of global goodness-of-fit indices were calculated. We used established criteria to interpret the fit of the estimated models (57,58): discrepancy chi-squared statistic (Chi^2)/degree of freedom (df) (ratio between 2 and 3 indicates good fit), Comparative Fit Index (CFI; values ≥0.90 indicate good fit), Tucker-Lewis Index (TLI; values ≥0.90 indicate good fit), Root Mean Square Error of Approximation (RMSEA; recommended value ≤0.06), and Standardized Root Mean Squared Residual (SRMR; recommended value <0.08). For comparison of models, we used Akaike information criterion (AIC) and Bayesian Information Criterion (BIC), for both of them a smaller value indicates a

better fit. Afterwards, we assessed reliability by McDonald's omega, here values above 0.70 indicate acceptable internal consistency (59)."

19. At line 298, the author refers to excluded cases as "data sets," which is unusual. From this point onward, the language requires careful revision by a native speaker, as there are several colloquial expressions that compromise the formal tone of the manuscript.
    - ➔ Thank you very much, this is absolutely correct and "data set" is not the right term in that context. We revised the according paragraph of the "Psychometric data analyses" subsection of the methods section and also improved on language:
      "We excluded cases if: 1) they were entirely empty, 2) had more than 30% missing AAS items (43), 3) participants did not meet the inclusion criteria, and/or 4) answered one of the two control questions incorrectly. These control questions were used to identify careless responses (44,45). We further screened cases for suspicious response behaviour (e.g., participant always chooses the same response option) and excluded suspicious cases after discussions within the study team."
      And we also revised the relevant paragraph of the "Psychometric evaluation" subsection of the results section:
      "Before analysis, 46 cases were excluded because they were empty (n=35), the inclusion criteria were not fulfilled (n=6), the second control question was incorrectly answered (n=3), and identical and suspicious responses for most of the scales (n=2). Data from 305 medical students were available for further analysis."

20. The manuscript does not clearly describe the estimation process for item difficulty.
    - ➔ We have added more details regarding item difficulty in section "Methods – Psychometric data analyses.": *"This involved examining response distribution, item difficulty (calculated by standardizing the mean to range from 0 to 1 by division by the maximum possible value of 5, recommended range 0.2 – 0.8) to assess floor and ceiling effects (item difficulties below 0.2 indicate a floor effect; item difficulties above 0.8 indicate a ceiling effect)."* Additionally, we added the following reference to this revised section: Döring N , Bortz J. Forschungsmethoden und evaluation. Wiesbaden: Springerverlag, 2006.

21. At line 342, a cut-off is mentioned, yet the methods section does not clarify which cut-off recommendations were followed.
    - ➔ Thank you very much for the detailed reading. Please see also our answer to your comment #18. We included the cut-off criteria in the revised methods section, but they were already included in table 6 of the results section before. This table is referred to in the according sentences of the results section (e.g., "Values of RMSEA and TLI did not meet cut-offs (65,66) (see Table 6)."). However, when working on this revision we realized, that the numbering of the last three tables were incorrect, we included the correct numbering in the revised manuscript (now: table 4, table 5 table 6).

22. Subsequently, the author analyzes item pairs without making explicit the rationale for their selection. If modification indices guided these choices, this should be explicitly stated.
    - ➔ Thank you for pointing on that, we tried to be more precise in the according paragraph of the results section (subsection "Psychometric evaluation / factor analysis") and included more information in the revised paragraph:
      "Additionally, we calculated modification indices for all item pairs and found the highest index for the combination of item 8 and 13. The χ^2 test statistic for freeing the covariance between those two items was 45.519 (degree of freedoms = 1). Upon further discussion within the study team, we identified a strong conceptual similarity between items 8 and 13, as both items express essentially the same statement using different wording."

23. Moreover, the writing from lines 345 to 354 lacks clarity and academic formality.
    - ➔ *According to your suggestion, we revised the according paragraph to increase clarity and formality:*

"Based on a discussion of these findings in conjunction with the item analysis for item 10, we decided to calculate an alternative model excluding item 10 (model 2). In model 2, factor loadings ranged from 0.437 (item 8) to 0.827 (item 9) (see Table 5). Model fits of model 2 were comparable to those of model 1, with RMSEA and TLI values remaining below the respective cut-off criteria. However, both AIC and BIC indicated a better fit for model 2 (see Table 6). Additionally, we calculated modification indices for all item pairs and found the highest index for the combination of item 8 and 13. The χ^2 test statistic for freeing the covariance between those two items was 45.519 (degree of freedoms = 1). Upon further discussion within the study team, we identified a strong conceptual similarity between items 8 and 13, as both items express essentially the same statement using different wording. Consequently, we calculated a third model (model 3) allowing the errors of item 8 and item 13 to correlate. Model 3 yielded factor loadings ranging from 0.418 to 0.819 (see Table 5) and demonstrated a better overall fit compared to models 1 and 2."

24. Table 3 presents three different models, one of which recommends the exclusion of an item. The authors should proceed with caution here, as they are suggesting modifications to an instrument based on a single sample of approximately 300 respondents. I strongly recommend revising the first discussion paragraph (lines 373–377), since it does not specify the target population for which the scale is intended. It should be restated that the measure is developed for medical students.
    - ➔ Thank you very much, we totally agree and revised the according paragraph as follows:
      "The original AAS is a brief instrument, which was developed and evaluated in 1983 in the United States for use by health educators helping students to explore their values concerning abortion (33). The instrument was originally developed and validated among high school and college students, but was later extended to a broader range of target populations."

25. In the discussion (lines 407–408), it is not clear where the information on sample motivation is introduced. This should either be explicitly stated in the text or referenced in a table for ease of reader comprehension.
    - ➔ Thank you very much for your suggestion, we carefully discussed in our study team. For the respective part of the discussion, we included findings on the students' preferred future field of work (see table 2) and their overall positive attitude toward abortions (see table 3). We discussed in the study team, that our specific study population might be rather interested in the topic of abortion care and therefore more motivated to take part in the study. Of course, motivation of study participants was not assessed within the survey. As you already stated above, representativity is also not given due to the recruitment strategy and only n=305 participant being included in data analysis. To address this issue as well, we revised the according paragraph of the discussion section:
      "In our sample of German medical students, most preferred to work in the field of gynecology and obstetrics later (see table 2) and most have a pro abortion attitude (see table 3). Based on the currently ongoing media-effective political and social discussions about abortion regulations in Germany, our sample might be rather motivated to participate in our study, which is followed by a self-selection bias. In addition, due to the recruitment strategy applied, we could not reach all German medical students and only n=305 students participated in the survey, meaning that our study population cannot be considered representative and no conclusions can be drawn about other groups.

26. In lines 412–415 of the discussion, there is no clear statement regarding the factor loading of item 10, which appears to be missing.
    - ➔ Thank you very much for the detailed reading. We indeed did not include information on the factor loading of item 10 in this part of the discussion section, thus we revised the according paragraph:
      "Item characteristics for the German AAS were satisfying for most items, except for item 10 (original: "People should not look down on those who choose to have abortions"). This item showed a low corrected item-total correlations, an unfavourable response distribution (demonstrating a ceiling effect), weak inter-item correlation, and a factor loading below the recommended threshold of 0.40 in the CFA. These results suggest that item 10 does not measure the underlying concept (50,51,69)."

27. Lines 418–422 would benefit from a clearer presentation of the scale's limitations. Freeing parameters is a post hoc solution in CFA—it allows error to be modeled, but the underlying issue with the instrument persists. Therefore, the authors need to provide stronger recommendations regarding the interpretation of results, the functioning of the scale, and its shortcomings. The recommendation to remove an item (lines 420–422) is problematic, particularly given (1) the limited sample size, and (2) the potential sample bias, which the authors themselves later acknowledge.

➔ We appreciate the reviewer's insightful comment and fully agree that freeing parameters in CFA represents a post hoc modification that does not resolve the underlying measurement limitations of the scale. In response, we have substantially revised this section to more clearly discuss the conceptual and psychometric shortcomings of the instrument and to provide a more cautious interpretation of our findings. We have also moderated our earlier recommendation to remove Item 10. Instead of proposing its exclusion, we now describe the 13-item version as a preliminary model that requires further psychometric validation in larger and more diverse samples before any firm conclusions can be drawn about the scale's optimal structure. These revisions ensure that our conclusions are appropriately cautious. Following changes were made to the revised discussion section:
"To conclude, the poor performance of item 10 likely reflects limited construct coverage rather than a mere statistical anomaly. After removing item 10 (model 2) and additionally allowing a correlation between item 8 and 13 based on modification indices and item's content (model 3), model 3 resulted in the best model fit with acceptable factor loadings (55,56,64). It has to be stated that allowing correlated errors between Items 8 and 13 was a data-driven adjustment. All performed model adjustments represent post hoc modifications and, together with limitations due to the limited sample size and the potential sample bias, should therefore be interpreted tentatively and with caution. Freeing parameters and removing items improve model fit statistically but do not fully resolve the underlying measurement issues of the scale. Consequently, we consider the findings preliminary and recommend that the 13-item version of the German AAS (without item 10) be subjected to further psychometric evaluation in larger and more diverse samples before any final modification of the instrument is adopted."

28. From lines 424–440, the manuscript discusses broader implications but fails to connect these to its own findings and to specify what is required for future applications of the AAS in the proposed context.

➔ We appreciate the reviewer's helpful observation. In response, we have substantially revised the discussion section to more clearly link the broader implications of our work to the empirical findings of the present study. Integrating also revisions we made according to your comment #27, the new version now explicitly refers to the psychometric results and explains how these findings inform future applications of the German AAS:
"However, physicians' attitudes toward abortion have been largely overlooked in empirical research. The successful translation and preliminary validation of the German version of the AAS highlights its potential as a culturally sensitive tool to evaluate attitudes towards abortions in sociocultural contexts where abortion remains ethically and politically sensitive. This adaptability is crucial for understanding how societal norms, laws, and individual beliefs intersect to shape attitudes towards abortions and abortion stigma in diverse populations. Our findings suggest that, while the overall factorial structure of the German AAS was supported, certain items (e.g., item 10 and the redundancy between item 8 and 13) require further psychometric refinement. These observations underline the importance of ensuring conceptual clarity and cultural appropriateness when applying the instrument in new populations. In the German context, the AAS may provide valuable insights into the attitudes of medical students and healthcare providers, helping to identify potential barriers to the provision of unbiased, person-centered abortion care. Understanding such attitudes early in medical training can inform the development of targeted educational interventions that address misconceptions and stigma (15,24,71). For future applications, replication of the AAS in larger and more diverse samples is needed, along with longitudinal studies to assess the stability of abortion attitudes over time and their sensitivity to social and legal change. Such efforts will strengthen the evidence base for using the AAS as a reliable measure to be used

by researchers, clinicians and policymakers to explore how community and professional attitudes toward abortion affect women's experiences, access to care, and decision-making. In this context, especially the assessment of experiences of stigma is relevant, particularly in contexts where restrictive laws and cultural norms contribute to feelings of shame, guilt, or fear among women seeking abortions."

29. At lines 463–464, the manuscript refers to the psychometric investigation as "good" and "satisfying," but these are subjective terms that do not adequately convey the findings. Furthermore, the discussion does not emphasize that this is only an initial step in adapting the scale, and that the results should be regarded as preliminary evidence of internal structure.
    - ➔ Thank you very much for your feedback on the conclusion section. According to your previously discussed recommendations and the respective revisions of the discussion section, we also revised the conclusion section and included the previously discussed aspects of preliminary results and limitations of the scale:
      "The German AAS is a brief measure that demonstrated high participant acceptance and acceptable psychometric properties. However, our findings indicate that certain items, particularly item 10 and the overlap between item 8 and item 13, require further refinement. Consequently, future validation should focus on the 13-item version of the scale, including examination of convergent and predictive validity in larger and more diverse samples. Despite these limitations, the German AAS shows potential as a tool for assessing attitudes towards abortion in Germany. This provides a foundation for identifying potential barriers in healthcare provision and informing the development of targeted educational and clinical interventions aimed at improving care for individuals facing unintended pregnancies."
      According to those changes, we also revised the conclusion section of the manuscripts abstract:
      "The German AAS is a brief measure that was accepted and demonstrated adequate psychometric properties. Suboptimal performance of some items indicated the need for further validation, particularly of a 13-item version. The AAS can improve evaluation of abortion attitudes in Germany. This may support identification of barriers in healthcare provision and improve care for unintentionally pregnant individuals."

30. Finally, given the small sample size, the preliminary nature of the results, and other methodological limitations, the manuscript might be more appropriately suited for submission to a smaller, specialized journal.
    - ➔ We appreciate the reviewer's concern regarding sample size and methodological considerations. We would like to note that the sample size is adequate for the conducted analyses and that the methodology employed is appropriate for a first validation study. We acknowledge, however, that abortion is a highly sensitive topic, strongly influenced by the legal, political, and sociocultural context of each country, as well as prevailing ethical and moral norms. This context adds complexity to the translation and cross-cultural application of the AAS, making direct comparability with other instruments or populations more challenging than for more general psychological constructs.
      Because of these complexities, we believe it is important to make these preliminary findings accessible to a broader readership rather than restricting them to a niche journal. The study provides the first German adaptation and psychometric evaluation of the AAS, contributing valuable insights into abortion attitudes among medical students and healthcare professionals. By disseminating these findings in a general, peer-reviewed journal, we aim to support future research, cross-cultural comparisons, and informed educational interventions while clearly acknowledging the preliminary and context-specific nature of our results.
